# Identification of QTL for Fiber Quality and Yield Traits Using Two Immortalized Backcross Populations in Upland Cotton

**DOI:** 10.1371/journal.pone.0166970

**Published:** 2016-12-01

**Authors:** Hantao Wang, Cong Huang, Wenxia Zhao, Baosheng Dai, Chao Shen, Beibei Zhang, Dingguo Li, Zhongxu Lin

**Affiliations:** 1 National Key Laboratory of Crop Genetic Improvement, College of Plant Science and Technology, Huazhong Agricultural University, Wuhan, Hubei, China; 2 State Key Laboratory of Cotton Biology, Institute of Cotton Research of CAAS, Anyang, Henan, China; 3 Institute of Crop Genetic and Breeding, College of Agriculture, Yangtze University, Jingzhou, Hubei, China; USDA-ARS Southern Regional Research Center, UNITED STATES

## Abstract

Two immortalized backcross populations (DHBCF_1_s and JMBCF_1_s) were developed using a recombinant inbred line (RIL) population crossed with the two parents DH962 and Jimian5 (as the males), respectively. The fiber quality and yield component traits of the two backcross populations were phenotyped at four environments (two locations, two years). One hundred seventy-eight quantitative trait loci (QTL) were detected including 76 for fiber qualities and 102 for yield components, explaining 4.08–17.79% of the phenotypic variation (PV). Among the 178 QTL, 22 stable QTL were detected in more than one environment or population. A stable QTL, *qFL-c10-1*, was detected in the previous F_2_ population, a RIL population in 3 environments and the current two BCF_1_ populations in this study, explaining 5.79–37.09% of the PV. Additionally, 117 and 110 main-effect QTL (M-QTL) and 47 and 191 digenic epistatic QTL (E-QTL) were detected in the DHBCF_1_s and JMBCF_1_s populations, respectively. The effect of digenic epistasis played a more important role on lint percentage, fiber length and fiber strength. These results obtained in the present study provided more resources to obtain stable QTL, confirming the authenticity and reliability of the QTL for molecular marker-assisted selection breeding and QTL cloning.

## Introduction

Cotton is the largest natural textile fiber provider and one of the important oil crops worldwide. Approximately 50 species have been discovered in the cotton genus (*Gossypium*), among which only four cultivated species (*G*. *herbaceum*, *G*. *arboreum*, *G*. *hirsutum*, and *G*. *barbadense*) could be used for fiber production [1]. Of the four cultivated species, *G*. *hirsutum*, also known as upland cotton, is widely cultivated because of its wide adaptability and high production, accounting for over 95% of the world’s cotton production [2].

Cotton fiber is an important raw material for the textile industry because of its softness and comfort; the products of cotton fiber are very popular [3]. With the stricter requirements of modern textile industry, the fiber quality of cotton is unable to meet the demands of the textile industry at present. Thus, the research on fiber development is particularly urgent. Cotton fiber is a spindly single cell derived from ovule epidermis. The development of cotton fiber is a complex process [4]. The molecular mechanism of fiber origination and elongation has been the research focus of scientists in cotton, and many novel genes related to fiber development have been detected [5–16]. For example, some genes related to fiber development based on a normalized fiber cDNA library have been verified using transgenic analysis in our laboratory [8,14,15,17–19]. Meanwhile, cotton breeders have been working on the improvement of lint yield. In the past several decades, the yield of cotton has been improved greatly, but this trend has been stagnant in recent years. The development of high yield and good-fiber-quality cultivars is the most urgent task for the cotton industry.

Fiber quality traits have been proven to be negatively correlated with yield traits in previous studies [3,20]. Although many genes related to fiber development and yield traits have been detected by reverse genetics, these genes are difficult to be used in breeding directly. The fast development of molecular maker technology has made it possible to map QTL for fiber quality and yield traits and to aggregate excellent genes controlling cotton yield and fiber quality using marker-assisted selection (MAS). The genome of upland cotton is complex and large [21], and the genetic background of upland cotton is narrow [22]. These reasons hinder the development of QTL mapping in upland cotton. At present, hundreds of QTL related to fiber quality and yield traits have been obtained using population genetics in upland cotton [3,23–30]. Some stable QTL related to yield traits were obtained, for example, *qBS-D8-1* and *qLP-D6-1* [31]. At the same time, many available QTL related to fiber length and fiber strength were also detected in previous studies, distributing on D3 and D11 [32], A1, D5 and D9 [24], A9 [3,23].

In this study, two immortalized backcross populations were developed from recombinant inbred lines (RILs) [3]. Both backcross populations were planted in four environments to detect stable QTL and confirm available QTL related to fiber quality and yield traits; thus, useful information will be provided for marker-assisted selection breeding and cloning candidate genes in the future.

## Materials and Methods

### Plant materials

A RIL population was developed by crossing *G*. *hirsutum* acc. DH962 and *G*. *hirsutum* cv. Jimian5 in a previous study [3]. Two backcross populations were developed in this research. The first backcross population contained 178 BCF_1_ hybrids (DHBCF_1_s), which were crossed between the RILs and DH962 (used as the male), and the second population contained 178 BCF_1_ hybrids (JMBCF_1_s), which were crossed between the RILs and Jimian5 (used as the male).

### Field experiments

DH962, Jimian5 and two backcross populations were planted on an experimental farm at Huanggang Normal College, Huanggang (30.45° N, 114.93° E), Hubei, China in 2013 (2013HG), 2014 (2014HG), and on an experimental farm at Yangtze University, Jingzhou (30.36° N, 112.15° E), Hubei, China in 2013 (2013JZ), 2014 (2014JZ). Each plot was 5-m long with 10 plants. A randomized block design was used to arrange the lines in the field. The data of the boll number per plant (BN) were collected in the middle of September, and twenty naturally opened bolls of each line were harvested in early October for fiber quality and yield investigation. Fiber qualities were measured using an HVI1000 Automatic Fiber Determination System at 20°C, and 65% relative humidity in the Institute of Cotton Research, Shihezi Academy of Agricultural Sciences, Xinjiang. Six yield and five fiber quality components were analyzed, including the seed cotton weight per boll (SCW), lint weight per boll (LW), lint percentage (LP), boll number per plant (BN), lint index (LI), seed index (SI), fiber length (FL, mm), fiber strength (FS, cN/tex), fiber length uniformity ratio (FU), fiber elongation (FE), and micronaire (MIC).

### Genotype analysis

A total of 634 primers were selected from Wang et al. [33] to genotype the RIL population [3], and a genetic map including 616 loci was constructed. The genotypes of the two backcross populations were deduced based on the genotypes of the RIL populations as the previous studies [34,35]. The genotypes of DHBCF_1_s (AA or AB) were deduced based on the cross of the genotypes of RILs (AA or BB) and DH962 (AA), and the genotypes of JMBCF_1_s (BB or AB) were deduced based on the cross of the genotypes of RILs (AA or BB) and Jimian5 (BB). If the genotypes were heterologous, we deduced that the genotypes of the BCF_1_ populations were heterologous.

### Data analysis and QTL detection

The differences in the phenotypic data between DH962 and Jimian5 were detected using t-test. The phenotypic data of the fiber quality and field components were analyzed using SPSS version 21.0 (SPSS, Chicago, IL, USA). The linkage map of an RIL population in a previous report was used for QTL mapping in the present study [3]. Additionally, the physical locations of the marker sequences were performed using a BLASTN search against the *G*. *hirsutum* (TM-1) genome [21] with an E-value cut-off of 1e^-10^. The composite interval mapping (CIM) method of Windows QTL Cartographer version 2.5 (http://statgen.ncsu.edu/qtlcart/WQTLCart.htm) was used to identify QTL for fiber quality and yield components of the two backcross populations. The mapping population type of the DHBCF_1_s and JMBCF_1_s populations were B1 and B2, respectively. The standard model (Model 6) was used to identify QTL action. The LOD threshold values were estimated by running 1,000 permutations to declare significant QTL for all of the traits [36]. The QTL with a LOD ≥ 2.5 was used to declare suggestive QTL, when the QTL’s confidence intervals overlapped in another environment or population with a LOD ≥ 2.0, it was considered to be a common QTL [37]. The main-effect QTL (M-QTL), digenic epistatic QTL (E-QTL) and their environmental interactions (QTL×environment, QE) of the two backcross populations were identified using two-locus analysis and the software ICIMapping 4.1 (http://www.isbreeding.net/software/?type=detail&id=18). The mapping population types of the DHBCF_1_s and JMBCF_1_s populations were P1BC1F1 and P2BC1F1, respectively. The model ICIM-ADD and ICIM-EPI were used for the analysis of M-QTL and E-QTL, respectively. The M-QTL with a LOD ≥ 2.5 was used to declare suggestive QTL, and a threshold of LOD ≥ 5.0 was used to declare the presence of E-QTL. QTL nomenclature was adapted according to the method in the previous report [38]. The graphic representation of the linkage map and QTL was drawn using MapChart V2.2 software [39].

## Results

### Fiber quality and yield traits under four environments

**Fig 1:**
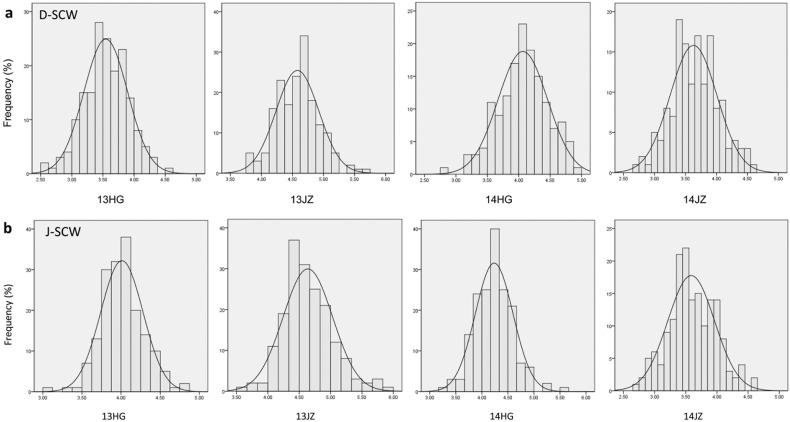
Frequency distribution of the SCW of the two BCF_1_ populations in four environments. a: SCW in DHBCF_1_s; b: SCW in JMBCF_1_s.

The trait data of fiber qualities and yield components of the parents and two BCF_1_ populations across four environments are shown in S1 Table. Significant differences between the parents were observed for most of the fiber and yield traits, except SI and LI. The parent DH962 was better in fiber qualities, and Jimian5 performed well in yield components. Skewness and kurtosis values showed that fiber quality and yield traits of the two BCF_1_ populations were almost approximately normally distributed (S1 Table; Fig 1; S1 Fig). For DHBCF_1_s, all the maximum phenotype data were larger than the parent DH962. In the JMBCF_1_s, the minimum phenotype data except FL in 2013HG was smaller than those in the parent Jimian5. These results showed that all traits performed transgressive segregation in the two BCF_1_ populations. Meanwhile, the average levels of the fiber quality traits of DHBCF_1_s were higher than those of JMBCF_1_s, and the average levels of the yield component traits of JMBCF_1_s were higher than those of DHBCF_1_s (S1 Table).

### Correlation between fiber quality and yield traits in two backcross populations

In DHBCF_1_s (S2 Table), SCW was significantly and positively correlated with LW, SI and MIC. LW was significantly and positively correlated with LP and MIC and was significantly and negatively correlated with FL and FS. LP was significantly and negatively correlated with FL, FU and FS. FL was significantly and positively correlated with FU and FS, and significantly and negatively correlated with MIC and FE. FU was significantly and positively correlated with FS, and significantly and negatively correlated with FE. MIC was significantly and positively correlated with FS and FE. All other correlations were neither significant nor stable. In JMBCF_1_s (S3 Table), SCW was significantly and positively correlated with LW, LI, SI and MIC. LW was significantly and positively correlated with LP and LI. LP was significantly and negatively correlated with SI, FL and FS. LI was significantly and positively correlated with SI and MIC. FL was significantly and positively correlated with MIC, FU, FS and FE. FE was significantly and positively correlated with FS and MIC.

Some stable correlations between different traits were obtained from the results of the two BCF_1_ populations. SCW was significantly and positively correlated with LW, SI and MIC. LW was significantly and positively correlated with LP. LP was significantly and negatively correlated with FL and FS. FL was significantly and positively correlated with FU and FS.

### QTL for fiber quality and yield component traits in two BCF_1_ populations

**Fig 2:**
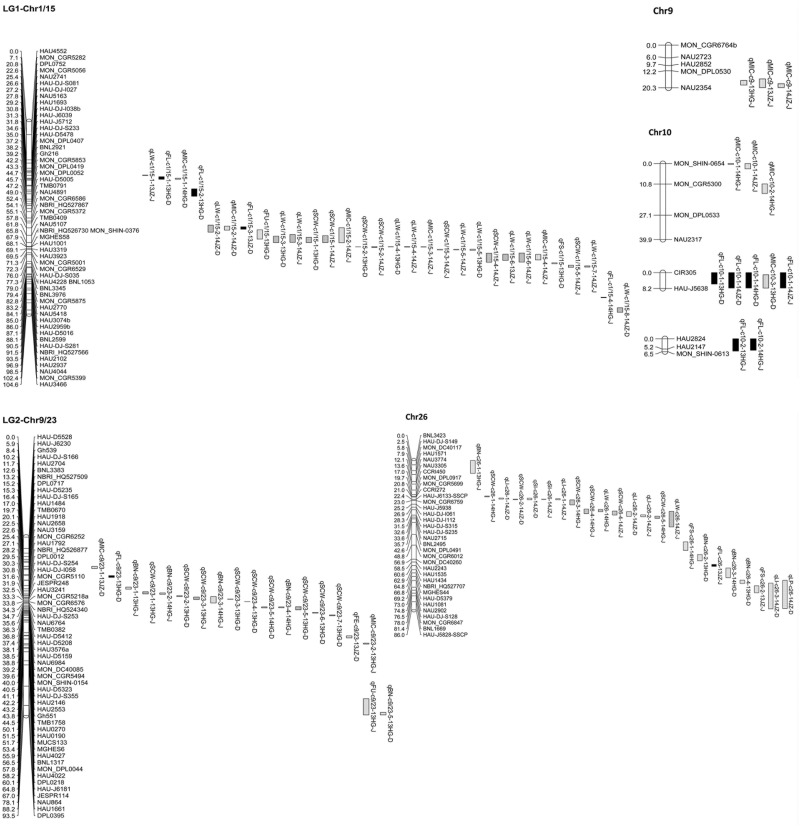
QTL mapping for fiber quality and yield component traits using two BCF_1_ populations in upland cotton.

A total of 178 QTL were detected on 23 chromosomes and 4 linkage groups in the two BCF_1_ populations, explaining 4.08–17.79% of the phenotypic variation (PV), with LOD scores ranging from 2.01 to 7.02 (S4 Table). Among the 178 QTL, 102 for six yield components and 76 for five fiber quality traits were identified in the two populations. Twenty-two (5 for SCW, 5 for LW, 1 for LP, 1 for SI, 2 for LI, 4 for FL, 3 for MIC, 1 for FS) of the 178 QTL were detected in more than one environment or population (Tables 1 and 2; Fig 2; S2 Fig).

**Table 1:** Stable QTL for yield component traits in the two backcross populations.

Trait	QTL	Marker interval	Position (cM)	LOD	R^2^	Additive	Population	Environment	Physical distance interval
SCW	qSCW-c1/15-1	TMB0409-MON_SHIN-0376	64.85	2.47	5.58%	0.17	DHBCF_1_s	13HG	A01 6270507–9869635
		TMB0409-MON_SHIN-0376	64.85	4.21	10.28%	0.24	JMBCF_1_s	14JZ	
	qSCW-c1/15-2	HAU1001-HAU3319	68.11	2.61	5.18%	0.16	DHBCF_1_s	13HG	A01 6331653–6403952
		HAU1001-HAU3319	68.11	3.43	7.30%	0.20	JMBCF_1_s	14JZ	
	qSCW-c9/23-3	MON_CGR5110-HAU3241	31.91	3.71	8.23%	-0.20	JMBCF_1_s	13HG	A09 64762371–65211179
		MON_CGR5110-HAU3241	32.56	2.01	4.08%	-0.14	DHBCF_1_s	13HG	
	qSCW-c9/23-5	TMB0382-HAU-D5208	36.8	2.96	6.53%	-0.21	DHBCF_1_s	14HG	D09 40566761–40742215
		TMB0382-HAU3576a	37.41	3.91	7.74%	-0.20	DHBCF_1_s	13HG	
	qSCW-c26-4	HAU-DJ-I112-HAU-DJ-S315	28.35	3.06	6.50%	0.20	JMBCF_1_s	14HG	D12 12212031–51124005
		HAU-DJ-I112-HAU-DJ-S315	30.35	2.46	5.42%	0.19	JMBCF_1_s	14JZ	
LW	qLW-c1/15-3	NAU5107-MON_SHIN-0376	63.85	3.16	7.64%	0.08	DHBCF_1_s	13HG	A01 6270824–7770095
		NAU5107-NBRI_HQ526730	63.85	4.3	10.51%	0.11	JMBCF_1_s	14JZ	
	qLW-c1/15-4	MGHES58-HAU3319	68.11	2.81	5.76%	0.07	DHBCF_1_s	13HG	A01 6331653–6403952
		HAU1001-HAU3319	68.11	2.7	5.92%	0.08	JMBCF_1_s	14JZ	
	qLW-c1/15-5	HAU3923-MON_CGR5001	69.56	2.8	6.08%	0.08	JMBCF_1_s	14JZ	A01 5552605–6331528
		HAU3923-MON_CGR5001	69.56	2.34	4.79%	0.06	DHBCF_1_s	13HG	
	qLW-c1/15-6	MON_CGR5001-HAU4228	73.33	2.45	5.82%	0.07	JMBCF_1_s	13JZ	A01 4545343–5552605
		MON_CGR5001-HAU4228	73.33	6.01	13.65%	0.13	JMBCF_1_s	14JZ	
	qLW-c26	HAU-DJ-I112-MON_DPL0491	32.57	3.92	7.44%	0.10	JMBCF_1_s	14JZ	D12 45872656–51124005
		HAU-DJ-I112-HAU-DJ-S315	28.35	2.2	4.63%	0.07	JMBCF_1_s	14HG	
LP	qLP-c11	HAU1721-HAU4514	63.66	2.95	9.70%	0.95	DHBCF_1_s	14JZ	A11 88514142–89310102
		HAU1721-HAU4514	67.66	2.7	5.78%	0.70	JMBCF_1_s	14JZ	
SI	qSI-c26	CCRI272-MON_CGR6759	22.4	3.46	7.90%	0.43	DHBCF_1_s	14JZ	D12 49834913–53157021
		CCRI272-MON_CGR6759	22.4	2.03	4.69%	0.27	JMBCF_1_s	14JZ	
LI	qLI-c26-1	CCRI272-HAU-J6133-SSCP	22.01	3.21	11.11%	0.33	DHBCF_1_s	14JZ	D12 49834913–53157021
		CCRI272-MON_CGR6759	22.4	3.47	7.39%	0.26	JMBCF_1_s	14JZ	
	qLI-c26-2	HAU-DJ-I112-HAU-DJ-S235	31.35	3	6.44%	0.26	DHBCF_1_s	14JZ	D12 45872656–51124005
		HAU-DJ-S315-HAU-DJ-S235	32.47	2.68	5.64%	0.23	JMBCF_1_s	14JZ	

**Table 2:** Stable QTL for fiber quality traits in the two backcross populations.

Trait	QTL	Marker interval	Position (cM)	LOD	R^2^	Additive	Population	Environment	Physical distance interval
FL	qFL-c2	BNL663-HAU-DJ4967	11.3	4.06	9.12%	0.67	DHBCF_1_s	13JZ	A02 82335820–82825609
		NAU2858-HAU-DJ4967	13.3	2.38	5.30%	0.50	JMBCF_1_s	14JZ	
	qFL-c10-1	CIR305-HAU-J5638	1.01	3.79	8.16%	0.69	DHBCF_1_s	13HG	A10 96187933–96657349
		CIR305-HAU-J5638	4.01	4.93	12.88%	0.86	DHBCF_1_s	14JZ	
		CIR305-HAU-J5638	6.01	6.29	17.79%	1.03	DHBCF_1_s	14HG	
		CIR305-HAU-J5638	7.01	3.94	9.22%	0.67	JMBCF_1_s	14JZ	
	qFL-c10-2	HAU2824-MON_SHIN-0613	0.01	5.49	10.68%	0.53	JMBCF_1_s	13HG	A10 100151919–100152739
		HAU2824-MON_SHIN-0613	2.01	2.95	6.81%	0.52	JMBCF_1_s	14HG	
	qFL-c21-2	HAU3033-HAU-DJ-S287	20.71	2.84	8.97%	0.64	JMBCF_1_s	14JZ	D11 60585668–64352278
		HAU3033-HAU-DJ-S287	26.71	2.15	5.34%	0.46	JMBCF_1_s	14HG	
MIC	qMIC-c1/15-2	MON_CGR5372-NAU5107	57.13	3.21	13.68%	0.22	DHBCF_1_s	14JZ	A01 6270507–9869635
		TMB0409-MON_SHIN-0376	64.85	5.95	13.70%	0.28	JMBCF_1_s	14JZ	
	qMIC-c9	MON_DPL0530-NAU2354	19.23	3.04	7.02%	0.17	JMBCF_1_s	13JZ	A09 73694867–74019922
		MON_DPL0530-NAU2354	20.23	2.95	6.03%	0.19	JMBCF_1_s	14JZ	
		MON_DPL0530-NAU2354	18.23	2.04	4.91%	0.11	JMBCF_1_s	13HG	
	qMIC-c10-1	MON_SHIN-0654-MON_CGR5300	0.01	2.62	5.71%	0.15	JMBCF_1_s	14HG	A10 541476–3063673
		MON_SHIN-0654-MON_CGR5300	0.01	2.3	4.63%	0.17	JMBCF_1_s	14JZ	
FS	qFS-c17-2	HAU-DJ-I091-BNL2441	41.24	2.93	7.38%	0.90	JMBCF_1_s	14HG	D07 26792784–46356023
		HAU-DJ-I091-BNL2441	43.24	2.41	5.72%	1.02	DHBCF_1_s	14JZ	

**SCW:** Twenty-nine QTL were detected on 11 chromosomes and 2 linkage groups, explaining 4.08–14.20% of the PV (S4 Table). Five stable QTL were identified (Table 1), and *qSCW-c1/15-1*, *qSCW-c1/15-2* and *qSCW-c9/23-3* were detected in different populations. *qSCW-c9/23-5* was detected in the DHBCF_1_s population in two years, and *qSCW-c26-4* was detected in the JMBCF_1_s population in two years.

**LW:** A total of 31 QTL were identified on 12 chromosomes and 2 linkage groups, explaining 4.63–16.27% of the PV (S4 Table). Five stable QTL were identified (Table 1), *qLW-c1/15-3*, *qLW-c1/15-4* and *qLW-c1/15-5* were detected in different populations. *qLW-c1/15-6* was identified in the JMBCF_1_s population in two years, explaining 5.82–13.65% of the PV. *qLW-c26* was also detected in the JMBCF_1_s population in two environments.

**LP:** Fifteen QTL associated with LP were detected in the two populations, explaining 5.22–12.54% of the PV (S4 Table). Seven QTL were identified in the DHBCF_1_s population, and 9 QTL were detected in the JMBCF_1_s population. *qLP-c11* was identified in the DHBCF_1_s and JMBCF_1_s populations in the same environment (2014JZ), explaining 5.78–9.70% of the PV (Table 1).

**BN:** Fourteen QTL were identified on 5 chromosomes and 1 linkage group in the two populations (S4 Table). Among the 14 QTL, 7 identified in each of the DHBCF_1_s and JMBCF_1_s populations. Five QTL were located on LG2-c9/23, and 4 were located on Chr26.

**SI and LI:** Five and eight QTL were detected for SI and LI, respectively (S4 Table). *qSI-c26* was identified in the DHBCF_1_s and JMBCF_1_s populations in the same environment (2014JZ), located between markers CCRI272 and MON_CGR6759 (Table 1). For LI, two common QTL were identified on Chr26 (Table 1), *qLI-c26-1* was identified in the two backcross populations in the same environment (2014JZ), explaining 7.39–11.11% of the PV. *qLI-c26-2* was also identified in the two populations in the same environment (2014JZ).

**FL:** Twenty-two QTL were detected on 11 chromosomes and 2 linkage groups, explaining 4.08–14.20% of the PV (S4 Table). Two stable QTL were identified on Chr10, the other two stable QTL were identified on Chr2 and Chr21, respectively (Table 2). *qFL-c2* was detected in the DHBCF_1_s and JMBCF_1_s populations in 13JZ and 14JZ, respectively, explaining 5.30–9.12% of the PV. *qFL-c10-1* was located between markers CIR305 and HAU-J5638 and was identified in two populations and three environments, explaining 8.16–17.79% of the PV, with LOD scores ranging from 3.79 to 6.29. *qFL-c10-2* was identified in the JMBCF_1_s population at two environments, explaining 6.81–10.68% of the PV. *qFL-c21-2* was identified in the JMBCF_1_s population at two environments, explaining 5.34–8.97% of the PV.

**FU:** Fourteen QTL for FU were identified on 9 chromosomes and 3 linkage groups (LG1-Chr1/15, LG2-Chr9/23, Chr9, Chr12, Chr14, Chr16, Chr17, Chr21, Chr22, Chr24, Chr25, LG3), 9 of which were located on the Dt genome (S4 Table). The 14 QTL for FU explained 4.98–9.05% of the PV, with the LOD scores ranging from 2.55 to 4.21, and no QTL was identified in more than one environment and population.

**MIC:** Twenty QTL were detected on 10 chromosomes and 2 linkage groups, explaining 4.63–13.70% of the PV (S4 Table). Three stable QTL were identified in more than one environment and population (Table 2). *qMIC-c1/15-2* was identified in the DHBCF_1_s and JMBCF_1_s populations in the same environment (2014JZ), explaining 13.68–13.70% of the PV. *qMIC-c9* was located between markers MON_DPL0530 and NAU2354 and was detected in the JMBCF_1_s population in three environments, explaining 4.91–7.02% of the PV. *qMIC-c10-1* was detected in the JMBCF_1_s population in two environments, explaining 4.63–5.71% of the PV.

**FS:** Seventeen QTL were identified on 9 chromosomes and 2 linkage groups, explaining 5.13–12.03% of the PV (S4 Table). *qFS-c17-2* was detected in the JMBCF_1_s and DHBCF_1_s population in 14HG and 14JZ, respectively, explaining 5.72–7.38% of the PV (Table 2).

**FE:** Three QTL were detected on 2 chromosomes and 1 linkage group, explaining 5.85–16.13% of the PV (S4 Table). *qFE-c22* was identified in the JMBCF_1_s population in 13JZ, explaining 16.13% of the PV, with an LOD score of 7.03.

### QTL and QE interactions in the two backcross populations

In total, 117 and 110 M-QTL and QEs were detected in the DHBCF_1_s and JMBCF_1_s populations, respectively (S5 Table). In the DHBCF_1_s population, 18, 15, 12, 4, 1, 7, 20, 9, 15 and 16 M-QTL and QEs for SCW, LW, LP, BN, SI, LI, FL, FU, MIC and FS were detected, with LOD scores ranging from 2.51 to 15.76. The M-QTL explained 0.0001–11.50% of the PV, and the QEs explained 0.03–7.14% of the PV. In the JMBCF_1_s population, 11, 14, 13, 6, 3, 26, 8, 17 and 12 M-QTL and QEs for SCW, LW, LP, BN, LI, FL, FU, MIC and FS were identified, with LOD scores ranging from 2.50 to 8.93. M-QTL explained 0.001–7.49% of the PV, and the QEs explained 0.0003–6.53% of the PV.

For E-QTL and QEs, 47 and 191 were identified in the DHBCF_1_s and JMBCF_1_s populations, respectively (S6 Table). In the DHBCF_1_s population, 2, 13, 1, 20, 2 and 9 E-QTL and QEs for SCW, LP, SI, FL, FU and FS were detected, with LOD scores ranging from 5.01 to 7.50. E-QTL explained 0.002–3.42% of the PV, and the QEs explained 0.00–5.28% of the PV. In the JMBCF_1_s population, 44, 2, 2, 109, 5, 10 and 19 E-QTL and QEs for LP, BN, SI, FL, FU, MIC and FS were detected, with LOD scores ranging from 5.01 to 9.65. E-QTL explained 0.002–6.61% of the PV, and the QEs explained 0.00–3.77% of the PV.

## Discussion

In the present study, a RIL population was crossed with the two parents (DH962 and Jimian5) as the males to construct two immortalized BCF_1_ populations. S1 Table shows that the average levels of fiber quality traits of DHBCF_1_s were higher than those of JMBCF_1_s, and the average levels of yield component traits of JMBCF_1_s were higher than those of DHBCF_1_s. The parents obviously affected the population performance. The differences in the fiber quality and yield component traits between the two BCF_1_ populations were useful for the QTL mapping on different traits [35,40].

In our previous studies, 33 QTL were detected using an F_2_ population crossed by DH962 and Jimian5 [33]. A RIL population developed by the same parents was phenotyped under 8 environments, identifying 134 QTL for fiber quality and yield traits [3]. In the present study, 178 QTL were detected in four environments using the two BCF_1_ populations. Using the F_2_ population, the RIL population and two BCF_1_ populations developed by the same parents could mutually increase the power of QTL detection, a finding that was consistent with previous studies in cotton [35,40]. Some new stable QTL were detected using the two BCF_1_ populations (Tables 1 and 2). For example, *qSCW-c1/15-1* and *qLW-c1/15-3* were detected in the two BCF_1_ populations and the same genome region. Two new QTL for FL, *qFL-c2* and *qFL-c21-2*, were identified. A stable QTL, *qMIC-c9*, was only detected in JMBCF_1_s for 3 environments. In addition, 5 of 33 QTL in the F_2_ population and 17 of the 134 QTL in the RIL population were verified in the two BCF_1_ populations (Table 3).

**Table 3:** Common QTL between the two backcross populations and the F_2_ or RIL population.

QTL	Marker interval	Position (cM)	LOD	R^2^	Additive	Population	Environment	Physical distance interval
qSCW-c9/23-1	NBRI_HQ526877-DPL0012	29.24	3.10	8.04%	-0.20	JMBCF1s	13HG	D09 37734904–39097842
**qSCW-c9/23-1**	**NBRI_HQ526877-DPL0012**	**29.24**	**2.80**	**5.74%**	**-0.12**	**RIL**	**11HG**	
	**NBRI_HQ526877-DPL0012**	**29.24**	**2.51**	**5.57%**	**-0.10**	**RIL**	**11JZ**	
qSCW-c9/23-3	MON_CGR5110-HAU3241	31.91	3.71	8.23%	-0.20	JMBCF1s	13HG	A09 64762371–65211179
	MON_CGR5110-HAU3241	32.56	2.01	4.08%	-0.14	DHBCF1s	13HG	
**qSCW-c9/23-2**	**MON_CGR5110-NBRI_HQ524340**	**33.30**	**3.20**	**5.92%**	**-0.12**	**RIL**	**11HG**	
	**HAU-DJ-I058-HAU-DJ-S253**	**33.30**	**3.99**	**7.82%**	**-0.12**	**RIL**	**11JZ**	
qSCW-c9/23-6	MON_DC40085-MON_SHIN-0154	39.6	2.80	5.64%	-0.17	DHBCF1s	13HG	D09 41639373–41995108
**qSCW-c9/23-5**	**HAU3576a-MON_CGR5494**	**39.25**	**3.27**	**6.46%**	**-0.11**	**RIL**	**11JZ**	
qLW-c24-3	HAU-DJ4940-HAU-DJ-S042	18.71	3.86	8.64%	0.09	JMBCF1s	13JZ	D08 17762712–19314578
**qLW-c24-2**	**HAU-DJ4940-HAU-DJ-S042**	**18.71**	**2.69**	**4.74%**	**-0.05**	**RIL**	**11HG**	
qLW-c26	HAU-DJ-I112-MON_DPL0491	32.57	3.92	7.44%	0.10	JMBCF1s	14JZ	D12 45872656–51124005
	HAU-DJ-I112-HAU-DJ-S315	28.35	2.20	4.63%	0.07	JMBCF1s	14HG	
**qLW-c26-5**	**HAU-DJ-I061-NAU2715**	**27.89**	**3.58**	**9.93%**	**0.07**	**RIL**	**11HG**	
	**HAU-DJ-I112-HAU-DJ-S315**	**31.35**	**2.53**	**5.54%**	**0.06**	**RIL**	**12HG**	
qLP-c17	HAU2688-HAU-DJ-I091	29.61	2.56	7.56%	-0.90	DHBCF1s	13HG	D07 46356023–50353380
**qLP-c17-2**	**HAU2688-HAU-DJ-S201**	**34.61**	**4.14**	**7.91%**	**-0.01**	**RIL**	**11HG**	
qLP-c25-1	TMB0313-HAU-DJ-I029	25.93	4.96	10.56%	1.40	JMBCF1s	13JZ	D06 10878971–36311206
**qLP-c25-1**	**BNL272-HAU-DJ-I029**	**30.91**	**2.93**	**5.98%**	**0.01**	**RIL**	**11HG**	
qLP-LG4	MON_CGR5796-CIR017	11.21	4.71	9.79%	1.56	JMBCF1s	13HG	-
**qLP-LG4**	**BNL2569-HAU1481**	**20.93**	**3.42**	**7.04%**	**-0.01**	**RIL**	**12HG**	
qBN-c5	HAU042-MON_CGR6760	0.01	2.60	6.31%	2.09	DHBCF1s	14HG	A05 86151819–86280526
**qBN-c5**	**HAU042-MON_CGR6760**	**4.01**	**2.94**	**5.91%**	**2.67**	**RIL**	**12HG**	
qSI-c26	CCRI272-MON_CGR6759	22.4	3.46	7.90%	0.43	DHBCF1s	14JZ	D12 49834913–57779295
	CCRI272-MON_CGR6759	22.4	2.03	4.69%	0.27	JMBCF1s	14JZ	
**qSI-c26**	**NAU3305-HAU-DJ-I061**	**17.92**	**4.26**	**13.19%**	**0.37**	**F**_**2**_		
qFL-c10-1	CIR305-HAU-J5638	1.01	3.79	8.16%	0.69	DHBCF1s	13HG	A10 96187933–96657349
	CIR305-HAU-J5638	4.01	4.93	12.88%	0.86	DHBCF1s	14JZ	
	CIR305-HAU-J5638	6.01	6.29	17.79%	1.03	DHBCF1s	14HG	
	CIR305-HAU-J5638	7.01	3.94	9.22%	0.67	JMBCF1s	14JZ	
**qFL-c10-1**	**CIR305-HAU-J5638**	**8.01**	**2.99**	**5.97%**	**0.40**	**RIL**	**11JZ**	
	**CIR305-HAU-J5638**	**0.01**	**4.83**	**9.14%**	**0.44**	**RIL**	**11HG**	
	**CIR305-HAU-J5638**	**8.01**	**5.30**	**11.07%**	**0.57**	**RIL**	**13HG**	
**qFL-c10**	**CIR305-TATAAG-1080**	**6.01**	**9.57**	**37.09%**	**1.16**	**F**_**2**_		
qFL-c10-2	HAU2824-MON_SHIN-0613	0.01	5.49	10.68%	0.53	JMBCF1s	13HG	A10 100151919–100152739
	HAU2824-MON_SHIN-0613	2.01	2.95	6.81%	0.52	JMBCF1s	14HG	
**qFL-c10-2**	**HAU2824-MON_SHIN-0613**	**6.25**	**4.01**	**7.72%**	**0.42**	**RIL**	**08HG**	
qFU-c21-1	NAU5389-HAU1467	0.01	4.21	9.05%	0.60	DHBCF1s	13HG	D11 2597022–2911281
**qFU-c21**	**NAU5389-HAU1467**	**2.01**	**2.87**	**7.63%**	**-0.20**	**RIL**	**08HG**	
qMIC-c1/15-1	HAU-DJ-I038b-HAU-J5712	31.27	3.13	7.54%	0.17	DHBCF1s	14HG	A01 38776461–90852809
**qMIC-c1/15-2**	**HAU1693-HAU-DJ-I038b**	**30.17**	**2.55**	**9.64%**	**0.10**	**RIL**	**09JZ**	
qMIC-c17	HAU2688-HAU-DJ-S201	35.24	3.60	7.36%	-0.18	JMBCF1s	14HG	D07 46356023–50353380
**qMIC-c17**	**HAU2688-HAU-DJ-S201**	**34.61**	**3.99**	**8.23**	**-0.12**	**RIL**	**13HG**	
qMIC-c22	NAU5046-MON_CER0050	53.01	3.67	8.70%	0.15	JMBCF1s	13HG	D04 14026153–45641917
**qMV-c22**	**HAU-D5527-TMB0206**	**37.67**	**5.38**	**12.24%**	**0.06**	**F**_**2**_		
qFS-c15	HAU4220-BNL830	15.72	4.58	12.03%	-1.32	JMBCF1s	13HG	D01 5821349–9663613
**qFS-c15**	**HAU4220-MON_CGR5826**	**0.01**	**4.05**	**8.41%**	**-0.61**	**RIL**	**09JZ**	
qFS-c17-1	HAU-DJ4982-HAU2688	10.92	2.54	5.13%	0.82	JMBCF1s	13HG	D07 50353530–51324951
**qFS-LG9-1**	**HAU-DJ4982-HAU2688a**	**38.19**	**4.13**	**18.88%**	**0.51**	**F**_**2**_		
qFS-c17-2	HAU-DJ-I091-BNL2441	41.24	2.93	7.38%	0.90	JMBCF1s	14HG	D07 26792784–46355807
	HAU-DJ-I091-BNL2441	43.24	2.41	5.72%	1.02	DHBCF1s	14JZ	
**qFS-c17**	**HAU2688-HAU-DJ-S201**	**35.24**	**4.93**	**9.82%**	**0.51**	**RIL**	**08HG**	
qFE-c22	MON_CER0050-HAU-D5397-SSCP	72.97	7.03	16.31%	-0.34	JMBCF1s	13JZ	D04 47584460–47894121
**qFE-c22**	**MON_CER0050-HAU-D5397-SSCP**	**71.97**	**4.96**	**11.92%**	**-0.03**	**RIL**	**08HG**	
	**MON_CER0050-HAU087**	**62.85**	**3.10**	**7.79%**	**0.04**	**RIL**	**11JZ**	
**qFE-c22-1**	**HAU-D5397-SSCP-HAU087**	**17.21**	**4.05**	**10.08%**	**-0.14**	**F**_**2**_		

Note: Common QTL in the F_2_ and RIL populations are in bold.

Regarding fiber length as one of the most indicators in fiber quality, the QTL *qFL-c10-1* was detected in the F_2_ population and RIL population in 3 environments and was detected in the two BCF_1_ populations in 3 environments, explaining 5.79–37.09% of the PV. A total of 470 QTL for fiber length distributed on 26 chromosomes have been collected in the Cotton QTL Database (http://www2.cottonqtldb.org:8081/index). Compared with these QTL, the QTL *qFL-c10-1* was only identified in our study; thus, the region between markers CIR305 and HAU-J5638 would be a novel important research focus for MAS and map-based cloning. *qFL-c10-2* was also an important locus for fiber length that was not only detected in the JMBCF_1_s population in two years and in the RIL population but was also identified as a major QTL in previous studies [23,25]. *qFE-c22* was detected as a major QTL in the RIL (*qFE-c22*) and F_2_ (*qFE-c22-1*) populations, respectively. In the yield component traits, *qSCW-c9/23-2* was detected in the RIL populations in two years and was verified as *qSCW-c9/23-3* in the two BCF_1_ populations. Three QTL related to lint percentage were also verified in the BCF_1_ population. These stable QTL of fiber quality and yield component traits identified in this research were more comprehensive and significant, which could be used for future fine mapping and gene cloning to promote molecular breeding in cotton.

Until now, the current release (Release 2.1) of the Cotton QTL Database collected 4,189 QTL from 132 publications of cotton. Many QTL distributed in the cotton whole genome revealed the complexity of the cotton genome and arduousness of QTL mapping in cotton. The identification of common QTL among the different studies is useful to confirm the authenticity and reliability of QTL. Compared with previous studies, some common QTL were detected according to the same markers on the same chromosomes. The QTL *qSCW-c21* was identified in a natural population by association analysis [41]. *qLW-c26* corresponded to the QTL *qLY-26* in an F_2:3_ population [42]. The QTL *qBN-Chr14-1* was detected as a stable QTL *qBNP-Chr14-1* in a RIL and a BC population crossed between upland cotton GX1135 and GX100-2 [40]. *qBN-c14-2* was detected as *qNB-D2-1* in a 4WC population [43]. Additionally, some stable QTL for fiber quality traits were obtained. *qFL-c10-2* was identified as a stable QTL in two studies [23,25]. *qFL-c25* was detected as the major QTL *qFL-C25-2* in a RIL population [44]. *qFU-c22* was the same as the QTL *qUI-c22* in a randomly mated recombinant inbred population [45]. Tan et al. (2015) obtained *qFM24*.*1* and *qFS07*.*1* using a high-density intraspecific genetic map [24], and they were the same as *qMIC-c24* and *qFS-c7* in the present study. Additionally, *qFS-c7* was also verified in F_2_ and RIL populations [46]. *qFS-c13-1* and *qFS-c13-2* were detected in an RIL population [44] and a natural population [47], respectively. The stable QTL *qFE-c22* was also confirmed as *qELO-c22* in the previous study [45]. The 12 common QTL detected by different populations confirmed the stability and veracity of these QTL, providing the resources for the fine mapping of this candidate QTL and developing functional markers for MAS.

After analysis by ICIM, 227 M-QTL were detected in the two BCF_1_ populations. Comparing the results of CIM and ICIM analysis, 94 QTL detected by CIM were verified in the ICIM analysis. The number of QTL detected by ICIM was more than that by CIM, and this phenomenon was consistent with that in previous studies [40,48]. For E-QTL, 238 E-QTL and QEs were obtained. This result showed that the E-QTL and QEs existed widely in the BCF_1_ populations, and epistasis played an important role in heterosis of the BCF_1_ populations [40,48]. The results of the E-QTL and QEs identified in the DHBCF_1_s and JMBCF_1_s populations showed that the number of E-QTL and QEs for LP, FL and FS were more than that of other traits, and digenic epistasis played a more important role in the heredity and expression of LP, FL and FS.

## Supporting Information

S1 Fig Frequency distribution of fiber quality and yield component traits of the two BCF_1_ populations in four environments. (TIF)

S2 Fig QTL mapping for fiber quality and yield component traits using two BCF_1_ populations in upland cotton. (TIF)

S1 Table Phenotypic value of fiber quality and yield component traits in the two BCF_1_ populations and parents. (XLS)

S2 Table Correlation analysis between fiber quality and yield component traits in DHBCF_1_s. (XLS)

S3 Table Correlation analysis between fiber quality and yield component traits in JMBCF_1_s. (XLS)

S4 Table QTL for fiber quality and yield component traits in the two BCF_1_ populations. (XLS)

S5 Table Main effects and environmental interactions detected for fiber quality and yield components in two BCF_1_ populations by inclusive composite interval mapping. (XLS)

S6 Table Epistatic effects and environmental interactions detected for fiber quality and yield traits in two BCF_1_ populations using two-locus analysis by inclusive composite interval mapping. (XLS)
